# Proteus effect avatar profiles: Associations with disordered gaming and activity levels

**DOI:** 10.1016/j.abrep.2024.100562

**Published:** 2024-08-02

**Authors:** Kaiden Hein, Tyrone L. Burleigh, Angela Gorman, Maria Prokofieva, Vasilis Stavropoulos

**Affiliations:** a School of Health and Biomedical Sciences, RMIT University, Melbourne, Australia; b Catholic Care Victoria, Victoria, Australia; c Institute for Health and Sport, Victoria University, Melbourne, Australia

**Keywords:** Internet Gaming Disorder, Avatar, Proteus Effect, Physical Activity, Digital

## Abstract

Gaming avatars can influence users’ attitudes and behaviors and manifest as the proteus effect. The present study examined proteus effect profiles among 571 gamers and their associations with disordered gaming and physical activity. Latent class analysis identified three profiles: non-influenced gamers, emotion-perception influenced gamers (highest proteus effect), and emotion-behavior influenced gamers (moderate proteus effect). The high proteus effect group exhibited significantly higher gaming disorder symptoms at baseline and 6 months compared to other profiles. Proteus effect profiles did not significantly differ in physical activity levels. However, higher disordered gaming and proteus effect predicted lower activity over time. The strong proteus effect group’s avatar immersion may increase gaming disorder risks. Minimal avatar influence for the non-influenced gamers appears protective. While proteus effect profiles do not directly relate to activity, amplified disordered gaming can reduce active lifestyles. Overall, findings demonstrate how avatars differentially affect gamers’ experiences and functioning through proteus-induced changes.

## Highlights

• Three avatar influence profiles identified: non-influenced, emotion-perception, emotion-behavior.
• Strongest avatar influence linked to higher disordered gaming symptoms over time.
• Avatar influence didn’t directly relate to physical activity, but indirectly reduced it.
• Negligible avatar influence protective against disordered gaming; strong bonds amplified risks.
• Optimal customization could promote healthy gaming, limit negative transference effects.

## 1 Introduction

Gaming is a worldwide phenomenon, approximately 67 % of the Australian community, where the present study was conducted, engage in gaming and over 2.2 billion people game globally (O’Farrell et al., 2020, Statista., 2023). Gaming can improve spatial ability, attentional capacity, and accuracy when multitasking (Podlogar & Podlesek, 2022). It can also enhance social connections and psychological wellbeing for many individuals (Bowman et al., 2022, Johannes et al., 2021). Gamification, which utilizes gaming principles has been applied in health and education settings to leverage the intrinsic motivation and engagement of gameplay (Sardi et al., 2017). Gamified health interventions (i.e., E-health) have shown positive outcomes including improved medication adherence for chronically ill patients (Sardi et al., 2017). However, excessive gaming has been linked to negative outcomes, such as reduced academic performance, social impairment, anxiety, stress, and depression (Ko et al., 2014, Pontes, 2017). As a result, the fifth edition of the diagnostic and statistical manual of mental disorders (DSM-5) has listed Internet Gaming Disorder (IGD) as a disorder warranting further research. Similarly, the International Classification of Diseases, 11th Revision (ICD-11), has included Gaming Disorder (American Psychiatric Association, 2023, World Health Organization, 2018).

IGD is characterized by impaired control and functioning due to excessive gaming. Individuals with IGD experience interference with routines, social interaction, family life, occupation, and self-care (Stevens et al., 2021). IGD prevalence is estimated at 1.96 % worldwide, with higher rates present throughout Asia (Stevens et al., 2021). Known risk factors include psychological conditions such as depression, anxiety disorders, and impulsivity (Kuss and Griffiths, 2012, Rho et al., 2017). Personality traits like avoidance and motivations of dissociation, and maladaptive coping strategies have also been linked to IGD risk (Kuss & Griffiths, 2012). Additionally, research indicates that males are 2.5 times more likely to develop IGD symptoms than females. Moreover, adolescents, children, and members of online groups have also been identified as particularly vulnerable to IGD (Pontes, 2017, Rho et al., 2017, Stevens et al., 2021). Furthermore, those with lower physical activity also have increased IGD susceptibility in emerging adulthood; however, active individuals appear to be more resilient to problematic IGD behaviors (Henchoz et al., 2016). Interestingly, participating in virtual reality exergames has been shown to boost fitness levels, reduce anxiety, and lessen problematic gaming behaviors, therefore acting as a protective factor against developing IGD behaviors (Maden et al., 2022).

Additionally, cultural capital (i.e., non-financial social assets that promote social mobility) within the metaverse (defined as immersive virtual shared spaces) and in-game flow are other aspects that have been thought to influence IGD behaviors (Hu et al., 2019, Korkeila and Hamari, 2020). Flow refers to a state of complete absorption and engagement in an activity, characterized by intense focus, loss of self-consciousness, and distorted sense of time. In gaming contexts, flow is often associated with optimal gaming experiences where players are fully immersed and challenged at a level matching their skills (Hu et al., 2019). Flow is shown to mediate the relationship between social gaming preference and addictive behaviors, with immersion occurring in both the game and resulting online interactions (Hu et al., 2019, Soutter and Hitchens, 2016). Indeed, the immersion achieved through customizing avatars can increase identification, motivation, flow, and potentially IGD risk (Korkeila & Hamari, 2020). However, some studies found conflicting results. While players often form stronger emotional connections to game characters, which may encourage IGD, these studies did not find games to be more immersive than other digital media. (Korkeila & Hamari, 2020; Van et al., 2018).

This has prompted research into gaming, IGD, and the relationship between users and their digital representations (Sibilla and Mancini, 2018, Mancini et al., 2019). An avatar is a digital representation of the gamer in a virtual world and serves as a conduit for self-expression and virtual identity formation (Ratan et al., 2020, Stavropoulos et al., 2020). This connection is often referred to as the user-avatar bond (UAB). The UAB represents the psychological attachment and identification a gamer develops with their digital representation (Ratan, 2012). It encompasses the extent to which gamers perceive their avatars as extensions of themselves, invest emotionally in their avatar, and the way in which the avatar is used to explore different aspects of their identity (Ratan, 2012). The UAB exists on a continuum from alignments to contrasts between user and avatar identities (Mancini and Sibilla, 2017, Sibilla and Mancini, 2018).

Research highlights that gamers express meaningful aspects of themselves by creating avatars that embody either idealized or actualized representations of themselves (Mancini and Sibilla, 2017, Sibilla and Mancini, 2018). Self-discrepancy theory suggests that idealized avatars can temporarily reduce the gap between the ideal and actual self, relieving psychological strain (Mancini and Sibilla, 2017, Van Looy et al., 2012). More specifically, four types of discrepancy profiles have been proposed in the literature: the negative hero, alter ego, idealized, and actualized avatars (Mancini & Sibilla, 2017). The idealized profile is an avatar with more socially desired traits than one's actual self, representing an aspirational version of the individual. The actualized profile embodies traits the gamer believes are attainable and realistic, reflecting their possible self. The negative hero profile explores anti-social traits, with “hero” referring to the avatar's role in the game rather than heroic qualities. The alter ego profile is an avatar that closely resembles the gamer’s current self, including both positive and negative traits and/or behaviors. This avatar allows gamers to express their actual self in a virtual environment, often highlighting aspects of their personality that they might not be able to express in real life. (Mancini & Sibilla, 2017). Some gamers also create utopian avatars who possess traits or skills that they aspire to but do not possess offline (Mancini et al., 2019). Evidence suggests that those viewing avatars as an ideal version of themselves identify more strongly with their avatars than users with socially undesirable avatars (Mancini and Sibilla, 2017, Mancini et al., 2019). Interestingly, non-humanoid avatars also readily allow bonds, indicating relationships stemming from characteristics beyond appearance (Stavropoulos et al., 2021).

High UAB identification is associated with increased IGD scores, especially for individuals with psychological distress like social anxiety or low self-esteem (Dengah and Snodgrass, 2020, Mancini et al., 2019). The perception of an improved but relatable avatar is thought to intensify IGD risk by reducing real world distress (Mancini et al., 2019), which may cause maladaptive coping mechanisms to form (Burleigh et al. 2019). In addition, customization of the avatar and presence also heightens intrinsic motivation, identification, and flow (Birk et al., 2016, Li and Lwin, 2016, Soutter and Hitchens, 2016). Interestingly, neuroimaging illustrates adolescents with IGD show medial prefrontal cortex activation when considering their avatars, suggesting a close interpersonal association (Choi et al., 2018). Thus, demonstrating how important the UAB is to understand the risks and protective factors of IGD. However, most studies utilize Massively Multiplayer Online Role-Playing Game (MMORPG; i.e., Role-playing games that are hosted on online servers with a focus on interacting with other players in a shared world; e.g., Final Fantasy XIV) players, limiting generalizability by homogenizing gamers (Szolin et al.,2022).

The Proteus effect (PE) is a psychological phenomenon that occurs when individuals adjust their attitudes and behaviors based on the characteristics of their avatars within virtual environments. More specifically, it refers to the observed changes in an individual's behavior and attitudes as a result of their digital self-representation. This effect highlights how the appearance and perceived traits of an avatar can lead to significant shifts in how a person thinks and acts in the game (Ash, 2016, Ratan, 2012, Stavropoulos et al., 2021). For example, gamers with more attractive avatars may feel more confident approaching others, while those with taller avatars might exhibit greater assertiveness compared to those with shorter avatars (Yee & Bailenson, 2007). Self-perception theory offers suggest that individuals infer their attitudes and behaviors by observing themselves in their avatar's role; this process leads to behavioral changes consistent with the avatar's characteristics (Liu, 2023, Bem, 1972). Additionally, priming offers another perspective on the PE; priming involves the activation of specific schemas or stereotypes in the user’s mind during gameplay. When players adopt avatars with certain traits, these characteristics can prime associated behaviors, leading to actions that align with the stereotypes of those traits (Ash, 2016).

The PE stands alongside game transfer phenomena as ways in which virtual experiences can influence real-world cognition and behavior. Game transfer phenomena describe the broader impact of gaming experiences on a player’s physiological and cognitive states beyond the virtual environment (Ortiz de Gortari & Gackenbach, 2021). While related to the effects of avatars on behavior, game transfer phenomena encompass a wider range of effects from gaming experiences. Taken together, these concepts underscore the complex, bidirectional relationship between users and their avatars (i.e., the UAB), affecting cognition, behavior, guilt, and the transference of avatar traits into the offline world (Stavropoulos et al., 2022).

As gamers become immersed in online games, problematic gaming may increase through heightened self-perception, deindividuation, and reinforcement of negative gaming emotions. The avatars' identities, through the lens of self-perception theory, extend their influence beyond personal perception, as gamers may infer their own attitudes and behaviors from their avatar's actions, affecting cognition and behavior both within and outside the virtual environment (i.e., the PE phenomena). For example, Reinhard and colleagues (2020) found participants walked at a lower or faster pace after using a virtual reality device, depending on their avatar’s age; highlighting that the manifestation of the PE can occur outside of the virtual environment. This connection results in a blurred distinction between avatar and self-identity, where the game's immersive qualities (e.g., avatar customization) can cause the gamers' offline behaviors to align with those of their avatars (Yee & Bailenson, 2007). Consequently, as the PE involves merging the avatar's characteristics with the user's actions and attitudes (thus strengthening the psychological and emotional investment in the avatar), individuals who experience the PE may experience excessive gaming patterns. However, a deeper understanding of how virtual identities influence player behavior is warranted (Stavropoulos et al., 2020).

A latent class analysis by Stavropoulos et al. (2020) categorized individuals who experienced the PE and examined their association with IGD. Three classes emerged: non-influenced gamers, perception/cognition-influenced gamers, and emotion/behavior-influenced gamers. Non-influenced gamers showed the lowest IGD symptoms, followed by perception/cognition-influenced gamers, while emotion/behavior-influenced gamers had the highest IGD associations including mood alteration and loss of control (Stavropoulos et al., 2020). However, Byrne et al. (2022) found no IGD and PE correlation, hypothesizing their diverse gamer sample explains the deviation. Although game transfer phenomena is associated with problematic gaming, the reverse is not always true (Ortiz de Gortari & Gackenbach, 2021). More research on UAB and related offline impacts is needed to understand IGD risks and inform protective interventions (Stavropoulos et al., 2022).

The PE health applications are promising but require additional study. For example, embodying young, fit avatars in virtual reality and exergames could see increased physical exertion, as self-perception theory suggests that individuals may align their behaviors with those of their avatars, thereby amplifying their engagement in activities. This alignment could then manifest the PE, causing individuals to carry over these increased activity levels into their offline lives (Kocur et al., 2021, Lin and Wu, 2021, Lin et al., 2021, Navarro et al., 2022). Indeed, research suggests that the PE can be observed through increased physical activity and effort offline, this is especially so when an avatar with a similar face to the individual is utilized (Lin and Wu, 2021, Lin et al., 2021, Navarro et al., 2022; Koncur et al., 2021). Whereas an individual's physical activity appears to decrease when their avatars are dressed more formally or have a stranger's face rather than their own (Navarro et al., 2022). Demonstrating that the embodiment of the avatar has the potential to modify behavior and cognition, as evidenced through the manifestation of the PE. However, research shows sex differences in how avatars impact exercise motivation, effort, and self-efficacy, with female participants being more persuaded than males to physically exert themselves and display increased self-efficacy when embodying normal sized, muscular, or athletic avatars (Lin et al., 2021, Lin and Wu, 2021, Lin and Wu, 2021, Lin et al., 2021). Avatar customization and embodiment can increase game persuasiveness by encouraging fulfilment of gender stereotypes through salient gendered avatar traits, though emotional bonds with avatars may only reduce related intentions rather than actual behaviors (Kang and Kim, 2020, Kim and Kim, 2016, Ratan and Sah, 2015). However, some studies find no greater persuasive impact of video games over other digital mediums regarding avatar embodiment and bonds (Van Looy et al. 2012).

Despite this, the PE has demonstrated reliability in *meta*-analysis with empirical evidence illustrating small to medium effect sizes (Ratan et al., 2020). It also offers an avatar specific construct to further analyze the UAB in a way that priming, and game transfer phenomena do not (Stavropoulos et al., 2021). The significance of the UAB, the negative consequences of IGD and its association with PE behaviors and decreased physical activity demonstrate the need for more research. Not only to add to the body of knowledge on IGD but also for its potential use in E-Health interventions that utilize gamification and increase physical activity and its protective value against IGD (Maden et al., 2022, Stavropoulos et al., 2021, Stavropoulos et al., 2022). In summary, the negative outcomes of IGD, its connection to physical inactivity and PE, and the potential for positive PE health applications highlight the need to investigate physical activity and PE as risk and protective factors for IGD development.

Thus, the present exploratory paper puts forward the following research questions:

(i) What are the different Proteus Effect profiles that can be identified through latent class analysis?

(ii) How do these identified Proteus Effect profiles relate to gaming disorder and physical activity:

a) Concurrently?

b) Over-time (6 months later)?

## 2 Method

### 2.1 Participants

A six-month longitudinal study investigated gaming patterns and demographics within an online community, collecting data from 627 participants at two separate intervals. After excluding preview-only responses (n = 7), spam (n = 19), bots (n = 1), those without consent (n = 12), invalid responses (n = 8), and incomplete responses (n = 15), the final sample consisted of 565 adolescents and adults (Mage = 29.3 years, SD=10.6; 50 % male, 45 % female, 4 % other gender). At the first time point, 55.3 % of participants reported being employed full-time, 36 % had an undergraduate degree, 73.6 % identified as heterosexual, 72.5 % indicated they were of Australian or English ancestry, 25.1 % lived with their family of origin, and 30.2 % were single. The inclusion criteria are that participants must be 12 years old and above, play online games with others, and have an avatar in their game world. The data, available online (see Stavropoulos, 2023), has been used in three previous studies (see Brown et al., 2024, Hein et al., 2024, Stavropoulos et al., 2023).

The maximum sampling error (N=571; 95 % confidence interval, Z=1.96) of 4.1 % adheres to recommended levels (+/- 7.1 %; Hill, 1998). Addressing statistical power, a G-power prior analysis computed Nmin = 74 for linear multiple regression R2 deviation from 0, modelling 15 predictors, an effect size of 0.15, and error probability (α) = 0.05 (Faul et al., 2007). There was a 48.8 % attrition rate, with 276 participants dropping out between study waves. Attrition/retention was inserted as an independent dummy coded variable (i.e., 0 = attrition, 1 = retention between waves one and two) to assess its associations with demographic characteristics. Attrition effects ranged from low to moderate for basic demographics and internet use information, including gender, sexual orientation, ancestry, romantic relationship involvement, education, employment, length of gaming engagement, and average daily gaming time during the week and weekend (Stavropoulos et al., 2023).

### 2.2 Materials

#### 2.2.1 Proteus effect Scale-Avatar identification in real life scale (PES-AIS; Van Looy et al., 2012)

The PES-AIS, a derivative of the Avatar Identification Scale (AIS) developed by Van Looy and colleagues (2012), assesses players' behavioral transference into the real world. Comprising six items loading onto a single factor measuring the influence of video games on behavior (e.g., “The choice of game character determines how I experience things in my real life”), participants respond on a five-point Likert scale (e.g., 1 = “strongly disagree” to 5 = “strongly agree”). Scores range from 6 to 30, with higher scores indicating a stronger proteus effect on behavior. Internal reliability of the instrument in the current study was robust (Cronbach α = 0.903, McDonald ω = 0.904).

#### 2.2.2 Internet gaming disorder Scale–Short-Form (IGDS9-SF; Pontes & Griffiths, 2015)

The IGDS9-SF is a standardized questionnaire that assesses Internet Gaming Disorder (IGD) according to the nine clinical criteria outlined in the latest edition of the DSM-5. Relating to the last 12 months, it contains nine items, each one measuring one of the nine criteria (e.g., I have significantly increased the amount of time I play games over last year). Participants respond on a 5-point Likert scale (e.g., 1 = “strongly disagree” to 5 = “strongly agree”). Scores range from 9 to 45, with higher scores indicating the possibility of internet gaming disorder being present. Internal reliability of the instrument in the current study was robust (Cronbach α = 0.846, McDonald ω = 0.858).

#### 2.2.3 Baseline physical activity

Measurement of physical activity was done with an actigraphy device (i.e., Fitbit devices [FD]), known for its validity in assessing moderate-to-vigorous physical activity (Feehan et al., 2018, Tully et al., 2014). The choice of FD aimed to minimize participant burden during extended wear periods, allowing participants to charge and sync devices at home, eliminating the need for multiple interactions with the research team throughout the study.

Participants were instructed to wear the FD on their non-dominant wrist continuously for seven days. The device utilized a microelectronic triaxial accelerometer to capture three-dimensional body motion (vertical, anteroposterior, and mediolateral), tracking steps, distance, energy expenditure, and active minutes (Feehan et al., 2018, Leung et al., 2022). The study focused on step counts as the primary physical activity indicator due to previous literature recommendations (Feehan et al., 2018, Fuller et al., 2020), comparability across individuals of varying body characteristics, and the device's high measurement accuracy (95–97 % accuracy for step counting when worn as directed; Evenson et al., 2015) compared to other indicators such as energy expenditure, known for its measurement inaccuracies (Feehan et al., 2018). Additionally, aggregating measurements (i.e., calculating mean scores) was deemed beneficial for enhancing measurement validity and reliability, particularly for extended periods of activity monitoring (Diaz et al., 2016, Liew et al., 2018). Thus, step counts were aggregated over seven days per time point in this study.

### 2.3 Procedure

The Human Ethics Research Committee of Victoria University (Australia) approved the study. Data collection occurred from November 2021 to November 2022. Recruitment was extensive, participants were gathered from various community sources. Interested individuals expressed their interest via a Qualtrics link distributed through social media (e.g., Facebook, Twitter), Victoria University websites, and digital forums (e.g., Discord). The study was open to adolescents and adults 12 years and older who volunteered anonymously. All participants read an information statement explaining their rights, the study's goals, and potential risks before giving informed consent. For adolescents aged 12–18, a parent or guardian's consent was obtained first, followed by the adolescent's assent. After reviewing the Plain Language Information Statement (PLIS) and consenting to participation, individuals underwent an in-person meeting where they received a Fitbit device and a questionnaire link accompanied by a unique numeric identifier. Researchers established individual accounts for participants based on their unique numeric identifiers, enabling remote data retrieval from Fitbit devices via the Fitbit website.

### 2.4 Statistical analyses

Data analysis was conducted with the tidyLPA package in R (Rosenberg et al., 2019) and the Statistical Package for the Social Sciences (SPSS). Latent class analysis (LCA) was employed to identify homogeneous subgroups (classes) of gamers based on their avatar-related behaviors assessed by the PES-AIS. LCA, a widely used latent variable modelling technique, identifies homogenous classes within a population based on the indicator of interest (Larose et al., 2016). LCA estimates various parameters (e.g., means, variances, covariances) simultaneously and allows for their comparison across classes, offering an accurate depiction of class characteristics. Multiple profile solutions (1 to 5 profiles) were compared across four different versions of parameter combinations (see Supplementary Table S1) to determine the optimal model fit.

Fit indices, including Akaike's Information Criterion (AIC), Approximate Weight of Evidence (AWE), Bayesian Information Criterion (BIC), Classification Likelihood Criterion (CLC), and Kullback Information Criterion (KIC), were evaluated to select the model with the best fit, with lower values indicating a better fit (Akogul & Erisoglu, 2017). The hierarchy of fit indices used were AIC, AWE, BIC, CLC, and KIC, based on literature recommendations (Akogul & Erisoglu, 2017). Additionally, entropy was used for model comparison and selection, with values ranging from 0 to 1, where 1 signifies perfect classification certainty (Celeux and Soromenho, 1996, Larose et al., 2016). An analysis of variance (ANOVA) assessed physical health variations across the different classes based on active step counts.

## 3 Results

Missing data were assessed using the missing completely at random test (MCAR; Zhang et al., 2019), indicating data were missing completely at random at the level of 1 % (χ2 = 41.5, df = 25, p = 0.02), while missing values averaged approximately 2 % (i.e., between 2.48 % and 2.12 %) considering the PES items (Field, 2017).

### 3.1 Model selection

A comparison of fit indices across the 20 models evaluated (four parameterizations multiplied by one to five classes) determined that the CIUP model with five classes exhibited the best fit according to AIC, AWE, BIC, CLC, and KIC (Akogul & Erisoglu, 2017; see Table 1). However, upon further examination of model fit statistics, including entropy and class size, issues related to class membership emerged. Notably, all models, except the CIUP model with five classes, exhibited average entropy values above the recommended threshold of 0.8 (Weller et al., 2020), suggesting inaccurate class delineation. This was further reflected in the small size of one class (class one), comprising less than 1 % of the sample. Therefore, considering all factors, the CIUP model with three classes was chosen as the optimal model based on fit indices, sample size, and classification quality, although a larger number of classes might have provided a statistically superior fit.

**Table 1:** Initial model testing.

Model	Profiles	AIC	BIC	AWE	CLC	KIC
Class Invariant	1	10483.321	10535.363	10645.405	10461.321	10498.321
Diagonal	2	8690.063	8772.462	8947.968	8653.957	8712.063
Parameterization	3	8364.076	8476.834	8717.790	8313.877	8393.076
(CIDP)	4	8378.055	8521.170	8827.653	8313.687	8414.055
	5	8117.896	8291.369	8662.986	8039.752	8160.896
Class Variant	1	10483.321	10535.363	10645.405	10461.321	10498.321
Diagonal	2	N.C	N.C	N.C	N.C	N.C
Parameterization	3	N.C	N.C	N.C	N.C	N.C
(CVDP)	4	N.C	N.C	N.C	N.C	N.C
	5	N.C	N.C	N.C	N.C	N.C
Class Invariant	1	8545.057	8662.151	8912.245	8493.057	8575.057
Unrestricted	2	8335.288	8482.740	8798.366	8269.114	8372.288
Parameterization	3	8156.332	8334.142	8715.056	8076.228	8200.332
(CIUP)	4	8137.890	8346.058	8792.902	8043.214	8188.890
	5	8017.270	8662.151	8767.765	7908.824	8075.270
Class Variant	1	8545.057	8662.151	8912.245	8493.057	8575.057
Unrestricted	2	N.C	N.C	N.C	N.C	N.C
Parameterization	3	N.C	N.C	N.C	N.C	N.C
(CVUP)	4	N.C	N.C	N.C	N.C	N.C
	5	N.C	N.C	N.C	N.C	N.C
Note: This table shows comparisons between different number of profiles for four possible combination of model parameters (including varying/fixed classes and varying/fixed covariances. Highlighted results (bold) indicate best model parameterization according to the best information criterion. Results showing N/C indicate that no convergence on a solution was possible. AIC=Akaike Information Criterion; BIC=Bayesian Information Criterion; AWE=Approximate Weight of Evidence Criterion; CLC=Classification Likelihood Criterion						

### 3.2 Size of classes

Descriptive methods were employed to calculate the size of each profile as valid percentages. The final count and proportion indicated that approximately 26.9 % of the sample belonged to class 1, 7.43 % to class 2, and 65.7 % to class 3 (see Table 2).

**Table 2:** Population share of the different PE gamers.

Profile	N	%
Class 1	152	26.9
Class 2	42	7.43
Class 3	371	65.7

### 3.3 Classification quality

The CIUP model with three classes demonstrated classification accuracy well above the recommended threshold of 0.76 (entropy = 0.95; Larose et al., 2016), indicating over 90 % class classification and robust membership across the three classes.

### 3.4 Classes across the indicator

**Fig. 1:**
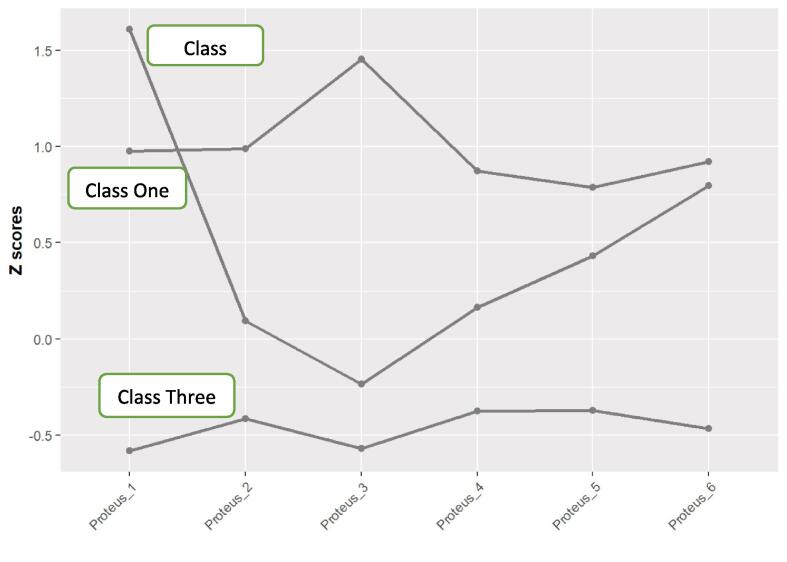
Standardised mean scores of the PES-AIS items for the different profiles. Note: Z scores represent standardised scores.

Within the three classes, class one exhibited standardized averages ranging from 0.787 to 1.45 standard deviations above the mean for all PES-IAS items. Class one displayed higher scores than one standard deviation above the mean on PE item 3 (“When I play with another character, I feel involved in a different way in my real life”). Consequently, individuals in class one were profiled as 'emotion and behavior influenced gamers'. Class two exhibited scores higher than one standard deviation above the mean for PE item one (“When I play with a different character, I feel different in my real life”) and below the mean for PE item three (“When I play with another character, I feel involved in a different way in my real life”). Therefore, individuals in class two were profiled as 'emotion and perception influenced gamers'. Lastly, class three demonstrated scores ranging from 0.371 to 0.582 below the mean on all PES items, leading to the profiling of individuals in this class as 'non-influenced gamers' (see Table 3 and Fig. 1).

**Table 3:** Standardised mean scores of the PES-AIS items for the different profiles.

Profile	Z PE_1	Z PE_2	Z PE_3	Z PE_4	Z PE_5	Z PE_6
Class 1 (EBIGs)	0.976	0.987	1.45	0.872	0.787	0.922
Class 2 (EPIGs)	1.61	0.094	−0.237	0.164	0.432	0.796
Class 3 (NIGs)	−0.582	−0.415	−0.569	−0.376	−0.371	−0.468

Note: Z scores represent standardised scores.

### 3.5 One-way ANOVA

Multiple one-way ANOVAs were run with the PE classes as the independent variable. Firstly, the one-way ANOVA on IGD at timepoint 1 across the three PE classes was significant, *F*(1, 558) = 32, *p* < 0.001. PE classes explained 5.1 % of the variance in IGD at timepoint one. Secondly, the one-way ANOVA on IGD at timepoint 2 across the three PE classes was also significant, *F*(1, 288) = 13.2, *p* < 0.001. PE classes explained 4.3 % of the variance in IGD at timepoint 2. Afterwards, two one-way ANOVAs were run, one on average weekly steps at timepoint 1 across three PE classes and the second using timepoint 2 average weekly steps. Both were not significant *F*(1, 452) = 1.13, *p* = 0.29; *F*(1, 252) = 0, *p* = 0.98, respectively.

Pairwise comparisons using the Bonferroni adjustment method were conducted to identify where the differences lie for the significant one-way ANOVAs. The ANOVA on IGD at timepoint 1 had significant comparisons between ‘emotion and behavior influenced gamers’ and ‘emotion and perception influenced gamers’ (*p* = 0.02); ‘emotion and behavior influenced gamers’ and ‘non-influenced gamers’ (*p* < 0.001) and was not significant between ‘emotion and perception influenced gamers’ and ‘non-influenced gamers’. The ANOVA on IGD at timepoint 2 had significant comparisons between ‘emotion and behavior influenced gamers’ and ‘non-influenced gamers’ (*p* < 0.001) and was not significant between ‘emotion and behavior influenced gamers’ and ‘emotion and perception influenced gamers’, ‘emotion and perception influenced gamers’ and ‘non-influenced gamers’.

### 3.6 Regression analysis

To check for linear associations between average weekly steps and PE and IGD, a multiple linear regression was conducted. Average weekly steps were set as the output variable and PE and IGD were set as predictor variables. Only data collected at timepoint one was utilized in this analysis. Multiple regression analysis revealed a significant model, *F*(2,441) = 3.78, *p* = 0.02. The total variance explained by the model when adjusting R squared was 1.2 %. IGD was found to be a unique significant predictor *t* = -2.61, *p* = 0.009. For every one-point increase of IGD, average weekly steps decreased by 98.7. While PE was not a significant predictor *t* = -0.13, *p* = 0.90.

## 4 Discussion

The present study had two main aims: To examine the occurrence of different PE profiles (i.e., a distinct class identified through latent class analysis, characterized by a unique pattern of responses in regard to their digital self-representation) and to assess how these profiles relate to UAB and physical activity concurrently and over time. Latent class analysis identified three distinct PE profiles among gamers: non-influenced gamers, emotion and behavior influenced gamers, and emotion and perception-influenced gamers. ‘Non-influenced gamers’ exhibited the lowest levels of PE, indicating their real-world attitudes and behaviors were minimally impacted by their avatars. In contrast, ‘emotion and behavior influenced gamers’ displayed the strongest PE, with avatars markedly influencing their emotions, perceptions, and real-world conduct. ‘Emotion and perception influenced gamers’ fell between these two extremes; their avatar-related effects centered primarily on shifted perceptions and thought processes rather than overt conduct changes.

Furthermore, PE profiles are differentially related to disordered gaming symptoms and physical activity. Specifically, ‘emotion and behavior influenced gamers’ showed significantly higher IGD behaviors at baseline and 6 months compared to the other profiles. However, there were no significant differences between classes in average daily step counts. Afterwards, a regression analysis demonstrated higher IGD was significantly associated with lower daily steps, while PE was not a significant predictor of daily steps.

### 4.1 Relationship between proteus effect profiles and user avatar bond

The identification of different PE profiles provides insight into the multifaceted relationships between avatars, game immersion, and offline functioning. As outlined in the introduction, more immersive avatars can augment players' sense of presence, thus heightening the PE (Birk et al., 2016). ‘Emotion and behavior influenced gamers’ in the current study exhibited the strongest PE, suggesting they experienced greater avatar embodiment and immersion that permeated into their offline attitudes and behaviors. This suggests that factors like customization and emotional bonds may intensify their avatars' persuasive pull (Li and Lwin, 2016, Soutter and Hitchens, 2016). In contrast, ‘non-influenced gamers’ displayed negligible avatar influence, which may indicate lower immersion with a weak sense of avatar connection. ‘Emotion and perception influenced gamers’ fell between these groups, with moderate PEs and resulting UAB.

The PE profiles align with established patterns in UABs and identity transference (Stavropoulos et al., 2020). Stronger bonds and blurred self-avatar identities appear to be associated with amplified PEs and higher IGD risks, as exhibited by ‘emotion and behavior influenced gamers’ (Choi et al., 2018, Stavropoulos et al., 2021). Their avatars' traits appear to transfer offline through demonstrating proteus-induced shifts. Weaker bonds among ‘non-influenced gamers’ conversely appear to limit the avatar identity permeability and offline effects. Again, ‘emotion and perception influenced gamers’ showed intermediate permeability and transference. These findings demonstrate how avatar connections shape online experiences and gaming risks through altered perceptions and embodiment.

### 4.2 Relationship between proteus effect profiles and gaming Disorder, and physical activity

The identification of PE profiles provides insight into how the avatar relates to disordered gaming symptoms and physical activity engagement. Notably, research has suggested that stronger emotional bonds with avatars can increase gaming immersion and potentially encourage problematic gaming behaviors (Burleigh et al., 2019, Mancini et al., 2019). The high PE exhibited by ‘emotion and behavior influenced gamers’ suggests strong UABs that may exacerbate maladaptive gaming through heightened presence and identity blurring. Indeed, the ‘emotion and behavior influenced gamers’ profile contained increases in baseline and longitudinal IGD scores which align with research linking greater UAB to amplified IGD risk (Stavropoulos et al., 2020, Stavropoulos et al., 2021). ‘Emotion and behavior influenced gamers’ cognitive, emotional, and behavioral permeability to their avatars appears to extend to problematic gaming behaviors and symptoms.

In contrast, ‘non-influenced gamers’ showed negligible PEs and had the lowest IGD scores out of the three profiles. Thus, it appears that having a weaker bond with their avatars attenuated immersion in gaming and resulting problematic behaviors (Stavropoulos, et al., 2020). This could be because ‘non-influenced gamers’ simply view their avatars as tools within the game, and thus the identity permeability and persuasive influence of the avatars is limited (Burleigh, et al., 2019). Their lack of emotional investment in the UAB suggests that gaming remained within healthy limits. These findings demonstrate how the depth of avatar customization and bonds can influence disordered gaming symptoms through controlled immersion. For ‘non-influenced gamers’, their UAB was shallow, minimizing disorder gaming risks and resulting in a low PE influence.

Interestingly, PE profiles did not relate to physical activity differences based on step counts. ‘Emotion and behavior influenced gamers’ showed the lowest activity levels, aligning with their gaming disorder score which has been linked to lower physical activities levels (Henchoz et al., 2016). However, significant variation between profiles was not evident, contrasting with research that suggests greater avatar bonds can reduce offline health behaviors through displacement (Stavropoulos et al., 2021). The lack of association suggests factors beyond PEs, like gaming motives and preferences, may better explain activity engagement. Nevertheless, higher IGD scores significantly predicted lower longitudinal steps, indicating excessive immersion could impede active lifestyles. Overall, these mixed findings warrant further examination into how avatar relationships motivate and inhibit physical pursuits. Additionally, customizing avatars to promote activity intentions could reveal promising interventions to counteract gaming's potential sedentary effects (Birk et al., 2016, Kang and Kim, 2020). For instance, one study found users with younger avatars were able to exert greater physical effort, highlighting the potential of avatar customization to enhance physical activity (Li et al., 2021). This evidence underscores the need to explore how gaming experiences can be leveraged to promote healthier behaviors.

The present findings demonstrate that the PE profiles, specifically ‘emotion and behavior influenced gamers’ susceptibility, provide valuable insights into IGD risks, however the differences in physical activity are less clear. While the PE profile and avatar do not appear to directly relate to activity levels, their amplification of disordered gaming appears to impact active behaviors over time. These findings highlight the complex interplay between gamers' PE profile, UAB, their gaming involvement, and related outcomes. Tailoring avatar customization to engender healthy immersion and motives can potentially mitigate obsessive symptoms while encouraging offline functioning. Further disentangling the nuances within avatar relationships can ultimately inform targeted interventions promoting controlled, rewarding play.

### 4.3 Limitations and Future research

Despite novel insights, there are a number of limitations which should be considered. The reliance on self-reports for gaming behaviors can involve response biases prone to social desirability concerns (Paulhus, 1991). Additionally, self-reported data may not have captured the implicit effects of the PE. Utilizing objective data (e.g., time spent customizing one’s avatar) may result in different associations being found. The adult sample also restricts generalizability to higher risk youth. Additionally, the two assessment timepoints spanned just six months. It should also be noted that the present paper was exploratory in nature, and thus no testable hypotheses were generated. Though, future research could build upon the present findings to develop and evaluate specific hypotheses.

Future longitudinal research should track adolescents/adults over longer follow-up periods to better understand how UABs evolve developmentally and impact online and offline functioning over time. Systematically manipulating avatar customization could isolate causal effects on disordered gaming and activity. While this study incorporated two timepoints, the relatively brief 6-month follow-up provides limited perspective on PE profile consistency. A longer follow-up could reveal different results by capturing potential effect decay or reinforcement, interactions with diverse real-world experiences, and individual differences in adaptation rates. However, extended follow-ups risk higher attrition, potentially compromising result validity. The six-month follow-up balanced these considerations, aiming to observe changes beyond immediate effects while mitigating practical constraints. Future research with varied follow-up durations would contribute to a more comprehensive understanding of the PE’s temporal trajectory, and the present study provides a foundation for such investigations. Ultimately, research clarifying how avatar relationships progress across at-risk age groups and influence experiences longitudinally, will provide essential insights to inform targeted, tailored interventions promoting healthy gaming and functioning.

### 4.4 Implications for research and practice

Our findings have implications for both researchers and practitioners in the fields of cyberpsychology and mental health. For researchers, this study underscores the importance of examining the long-term psychosocial effects of avatar use. Future longitudinal studies should investigate how gamer-avatar relationships evolve over time and influence offline behaviors, potentially uncovering mechanisms for early intervention in problematic gaming. For mental health professionals, our results highlight the need to consider clients' relationships with their gaming avatars when assessing and treating IGD. Therapeutic interventions might focus on helping clients distinguish between their virtual and real-world identities, possibly by strengthening real-world social connections and self-concept (Lemenager et al., 2020).

### 4.5 Conclusion

This study identified distinct subgroups of gamers based on their PE profiles in relation to their avatars. ‘Emotion and behavior influenced gamers’ experienced the strongest avatar-related shifts in emotions, cognitions, and offline conduct. Their greater immersion and identification likely contributed to amplified effects that have been associated with increased disordered gaming scores. In contrast, ‘non-influenced gamers’ displayed minimal avatar influence and gaming disorder scores. Moreover, PE profiles did not appear to be significantly associated with physical activity levels between profiles, however IGD scores were found to be higher in profiles that were highly influenced by their avatars, like ‘emotion and behavior influenced gamers’, which may be indirectly related to reduced activity engagement over time. These findings highlight how avatars differentially permeate gamers’ online experiences and offline functioning through proteus-induced changes. Additional research is needed to extend these insights across diverse age groups and explore causality between PEs and disordered gaming over time. Uncovering optimal avatar bonds that maximize immersion yet constrain negative transference can ultimately inform protective interventions.

Compliance with Ethical Standards.

Informed consent was obtained from all participants included in the study.

All procedures in studies involving human participants were performed in accordance with the ethical standards of the Victoria University Human Research Ethics Committee [HRE21-044], the Department of Education and Training of The Victorian State Government, Australia [2022_004542] and the Melbourne Archdiocese of Catholic Schools [1179].

Funding:

While working on the manuscript, Dr. Vasileios Stavropoulos was supported by a grant from the 10.13039/501100000923Australian Research Council, Discovery Early Career Researcher Award, 2021, number DE210101107.

Ethical Standards – Animal Rights:

All procedures performed in the study involving human participants were in accordance with the ethical standards of the institutional and/or national research committee and with the 1964 Helsinki declaration and its later amendments or comparable ethical standards. This article does not contain any studies with animals performed by any of the authors.

Confirmation Statement:

Authors confirm that this paper has not been either previously published or submitted simultaneously for publication elsewhere.

## CRediT authorship contribution statement

**Kaiden Hein:** Writing – review & editing, Writing – original draft, Project administration, Methodology, Investigation, Formal analysis, Data curation, Conceptualization. **Tyrone L. Burleigh:** Writing – review & editing. **Angela Gorman:** Resources. **Maria Prokofieva:** Formal analysis, Data curation. **Vasilis Stavropoulos:** Writing – review & editing, Writing – original draft, Supervision, Methodology, Investigation, Formal analysis, Data curation, Conceptualization.

## Declaration of competing interest

The authors declare that they have no known competing financial interests or personal relationships that could have appeared to influence the work reported in this paper.

## Data Availability

PLease see attached file step

## References

[b0005] Akogul S., Erisoglu M. (2017). An approach for determining the number of clusters in a model-based cluster analysis. Entropy.

[b0010] American Psychiatric Association (2023).

[b0015] Ash E. (2016). Priming or Proteus Effect? Examining the effects of avatar race on in-game behavior and post-play aggressive cognition and affect in video games. Games and Culture.

[b0020] Bem D.J., Berkowitz L. (1972).

[b0025] Birk M.V., Atkins C., Bowey J.T., Mandryk R.L. (2016). in *Proceedings of the 2016 CHI Conference on Human Factors in Computing Systems*.

[b0030] Bowman N.D., Rieger D., Tammy Lin J.H. (2022). Social video gaming and well-being. Current Opinion in Psychology.

[b0035] Brown T., Burleigh T.L., Schivinski B., Bennett S., Gorman-Alesi A., Blinka L., Stavropoulos V. (2024). Translating the user-avatar bond into depression risk: A preliminary machine learning study. Journal of Psychiatric Research.

[b0040] Burleigh T.L., Griffiths M.D., Sumich A., Stavropoulos V., Kuss D.J. (2019). A systematic review of the co-occurrence of gaming disorder and other potentially addictive behaviors. Current Addiction Reports.

[b0045] Byrne S., Allen A., Stavropoulos V., Kannis-Dymand L. (2022). Problematic gaming: The role of desire thinking, metacognition, and the Proteus Effect. Behaviour and Information technology.

[b0050] Celeux G., Soromenho G. (1996). An entropy criterion for assessing the number of clusters in a mixture model. Journal of classification.

[b0055] Choi E.J., Taylor M.J., Hong S.B., Kim C., Kim J.W., McIntyre R.S., Yi S.H. (2018). Gaming-addicted teens identify more with their cyber-self than their own self: Neural evidence. Psychiatry Research: Neuroimaging.

[b0060] Dengah F.H.J., Snodgrass J.G. (2020). Avatar creation in video gaming: Between compensation and constraint. Games for Health Journal.

[b0065] Diaz K.M., Krupka D.J., Chang M.J., Shaffer J.A., Ma Y., Goldsmith J., Schwartz J.E., Davidson K.W. (2016). Validation of the Fitbit One® for physical activity measurement at an upper torso attachment site. BMC Research Notes.

[b0070] Evenson K.R., Goto M.M., Furberg R.D. (2015). Systematic review of the validity and reliability of consumer-wearable activity trackers. International Journal of Behavioral Nutrition and Physical Activity.

[b0075] Faul F., Erdfelder E., Lang A.G., Buchner A. (2007). G*Power 3: A flexible statistical power analysis program for the social, behavioral, and biomedical sciences. Behavior Research Methods.

[b0080] Feehan L.M., Geldman J., Sayre E.C., Park C., Ezzat A.M., Yoo J.Y., Hamilton C.B., Li L.C. (2018). Accuracy of Fitbit devices: Systematic review and narrative syntheses of quantitative data. JMIR mHealth and uHealth.

[b0085] Field A. (2017).

[b0090] Fuller D., Colwell E., Low J., Orychock K., Tobin M.A., Simango B., Taylor N.G. (2020). Reliability and validity of commercially available wearable devices for measuring steps, energy expenditure, and heart rate: Systematic review. JMIR mHealth and uHealth.

[b0095] Hein K., Conkey-Morrison C., Burleigh T.L., Poulus D., Stavropoulos V. (2024). Examining how gamers connect with their avatars to assess their anxiety: A novel artificial intelligence approach. Acta Psychologica.

[b0100] Henchoz Y., Studer J., Deline S., N’Goran A.A., Baggio S., Gmel G. (2016). Video gaming disorder and sport and exercise in emerging adulthood: A longitudinal study. Behavioral Medicine.

[b0105] Hill, R. (1998). What sample size is “enough” in internet survey research. *Interpersonal Computing and Technology: An electronic journal for the 21st century*, *6*(3-4), 1-12.

[b0110] Hu E., Stavropoulos V., Anderson A., Scerri M., Collard J. (2019). Internet gaming disorder: Feeling the flow of social games. Addictive Behaviors Reports.

[b0115] Johannes N., Vuorre M., Przybylski A.K. (2021). Video game play is positively correlated with well-being. Royal Society Open Science.

[b0120] Kang H., Kim H.K. (2020). My avatar and the affirmed self: Psychological and persuasive implications of avatar customization. Computers in Human Behavior.

[b0125] Kim H.K., Kim S.H. (2016). Understanding emotional bond between the creator and the avatar: Change in behavioral intentions to engage in alcohol-related traffic risk behaviors. Computers in Human Behavior.

[b0130] Ko C.H., Yen J.Y., Chen S.H., Wang P.W., Chen C.S., Yen C.F. (2014). Evaluation of the diagnostic criteria of Internet gaming disorder in the DSM-5 among young adults in Taiwan. Journal of Psychiatric Research.

[b0135] Kocur M., Habler F., Schwind V., Wozniak P.W., Wolf C., Henze N. (2021). Physiological and perceptual responses to athletic avatars while cycling in virtual reality. Conference on Human Factors in Computing Systems - Proceedings..

[b0140] Korkeila H., Hamari J. (2020). Avatar capital: The relationships between player orientation and their avatar’s social, symbolic, economic and cultural capital. Computers in Human Behavior.

[b0145] Kuss D.J., Griffiths M.D. (2012). Internet gaming addiction: A systematic review of empirical research. International Journal of Mental Health and Addiction.

[b0150] Larose C., Harel O., Kordas K., Dey D.K. (2016). Latent class analysis of incomplete data via an entropy-based criterion. Statistical Methodology.

[b0155] Liew L.W.L., Stavropoulos V., Adams B.L.M., Burleigh T.L., Griffiths M.D. (2018). Internet Gaming Disorder: The interplay between physical activity and user–avatar relationship. Behaviour & Information Technology.

[b0160] Lemenager T., Neissner M., Sabo T. (2020). “Who Am I” and “How Should I Be”: A Systematic Review on Self-Concept and Avatar Identification in Gaming Disorder. Curr Addict Rep.

[b0165] Leung W., Case L., Sung M.C., Jung J. (2022). A meta-analysis of Fitbit devices: Same company, different models, different validity evidence. Journal of Medical Engineering & Technology.

[b0170] Li B.J., Lwin M.O. (2016). Player see, player do: Testing an exergame motivation model based on the influence of the self avatar. Computers in Human Behavior.

[b0175] Lin J.H.T., Wu D.Y. (2021). Exercising with embodied young avatars: How young vs. older avatars in virtual reality affect perceived exertion and physical activity among male and female elderly individuals. Frontiers in psychology.

[b0180] Lin J.H.T., Wu D.Y., Yang J.W. (2021). Exercising with a six pack in virtual reality: Examining the proteus effect of avatar body shape and sex on self-efficacy for core-muscle exercise, self-concept of body shape, and actual physical activity. Frontiers in psychology.

[b0185] Liu Y. (2023). The Proteus Effect: Overview, reflection, and recommendations. Games and Culture.

[b0190] Maden Ç., Bayramlar K., Arıcak O.T., Yagli N.V. (2022). Effects of virtual Reality-Based Training and aerobic training on gaming disorder, physical activity, physical fitness, and anxiety: A randomized, controlled trial. Mental Health and Physical Activity.

[b0195] Mancini T., Sibilla F. (2017). Offline personality and avatar customization. Discrepancy profiles and avatar identification in a sample of MMORPG players. Computers in Human Behavior.

[b0200] Mancini T., Imperato C., Sibilla F. (2019). Does avatar’s character and emotional bond expose to gaming addiction? Two studies on virtual self-discrepancy, avatar identification and gaming addiction in massively multiplayer online role-playing game players. Computers in Human Behavior.

[b0205] Navarro J., Peña J., Cebolla A., Baños R. (2022). Can avatar appearance influence physical activity? User-avatar similarity and proteus effects on cardiac frequency and step counts. Health Communication.

[b0210] Ortiz de Gortari A.B., Gackenbach J. (2021). Game transfer phenomena and problematic interactive media use: Dispositional and media habit factors. Frontiers in Psychology.

[b0215] O’Farrell D., Baynes K., Pontes H., Griffiths M., Stavropoulos V. (2020). Depression and disordered gaming: Does culture matter?. International Journal of Mental Health and Addiction.

[b0220] Podlogar N., Podlesek A. (2022). Comparison of mental rotation ability, attentional capacity and cognitive flexibility in action video gamers and non-gamers. Cyberpsychology.

[b0225] Paulhus, D. L. (1991). Measurement and control of response bias. *Measures of Personality and Social Psychological Attitudes*, 17–59. https://doi. org/10.1016/B978-0-12-590241-0.50006-X.

[b0230] Pontes H.M. (2017). Investigating the differential effects of social networking site addiction and Internet gaming disorder on psychological health. Journal of Behavioral Addictions.

[b0235] Pontes H.M., Griffiths M.D. (2015). Measuring DSM-5 internet gaming disorder: Development and validation of a short psychometric scale. Computers in human behavior.

[b0240] Ratan, R. A. (2012). Self-presence, explicated: Body, emotion, and identity extension into the virtual self. In R. Luppicini (Ed.), *Handbook of Research on Technoself* (pp. 322–336). IGI Global 10.4018/978-1-4666-2211-1.ch018.

[b0245] Ratan R., Beyea D., Li B.J., Graciano L. (2020). Avatar characteristics induce users’ behavioral conformity with small-to-medium effect sizes: A meta-analysis of the proteus effect. Media Psychology.

[b0250] Ratan R., Sah Y.J. (2015). Leveling up on stereotype threat: The role of avatar customization and avatar embodiment. Computers in Human Behavior.

[b0260] Rho M.J., Lee H., Lee T.-H., Cho H., Jung D.J., Kim D.-J., Choi I.Y. (2017). Risk factors for internet gaming disorder: Psychological factors and internet gaming characteristics. International Journal of Environmental Research and Public Health.

[b0265] Rosenberg J.M., Beymer P.N., Anderson D.J., Van Lissa C.J., Schmidt J.A. (2019). tidyLPA: An R package to easily carry out latent profile analysis (LPA) using open-source or commercial software. Journal of Open Source Software.

[b0270] Sardi L., Idri A., Fernández-Alemán J.L. (2017). A systematic review of gamification in e-Health. Journal of Biomedical Informatics.

[b0275] Sibilla F., Mancini T. (2018). I am (not) my avatar: A review of the user-avatar relationships in massively multiplayer online worlds. Cyberpsychology.

[b0280] Soutter A.R., Hitchens M. (2016). The relationship between character identification and flow state within video games. Comput. Hum. Behav..

[b0285] Statista. (2023). Digital media market: Number of users of video games worldwide 2017-2027. https://www.statista.com/statistics/748044/number-video-gamers-world/.

[b0295] Stavropoulos V., Pontes H.M., Gomez R., Schivinski B., Griffiths M. (2020). Proteus effect profiles: How do they relate with disordered gaming behaviors?. Psychiatric Quarterly.

[b0300] Stavropoulos V., Ratan R., Lee K.M. (2022). User-avatar bond: Risk and opportunities in gaming and beyond. Frontiers in Psychology.

[b0305] Stavropoulos V., Rennie J.M., Gomez R., Griffiths M.D. (2021). Understanding the relationship between the Proteus effect, immersion, and gender among World of Warcraft players: An empirical survey study. Behaviour & Information Technology.

[b0310] Stavropoulos V., Zarate D., Prokofieva M., Van de Berg N., Karimi L., Gorman Alesi A., Griffiths M.D. (2023). Deep learning (s) in gaming disorder through the user-avatar bond: A longitudinal study using machine learning. Journal of Behavioral Addictions.

[b0315] Stevens M.W., Dorstyn D., Delfabbro P.H., King D.L. (2021). Global prevalence of gaming disorder: A systematic review and meta-analysis. Australian & New Zealand Journal of Psychiatry.

[b0320] Szolin K., Kuss D., Nuyens F., Griffiths M. (2022). Gaming Disorder: A systematic review exploring the user-avatar relationship in videogames. Computers in Human Behavior.

[b0325] Tully M.A., McBride C., Heron L., Hunter R.F. (2014). The validation of Fitbit ZipTM physical activity monitor as a measure of free-living physical activity. BMC Research Notes.

[b0330] Van Looy J., Courtois C., De Vocht M., De Marez L. (2012). Player identification in online games: Validation of a scale for measuring identification in MMOGs. Media Psychology.

[b0335] Weller B.E., Bowen N.K., Faubert S.J. (2020). Latent class analysis: A guide to best practice. Journal of Black Psychology.

[b0340] World Health Organization (2018). ICD-11 beta draft: Gaming disorder. Retrieved June 2nd, 2023, from: https://www.who.int/features/qa/gaming-disorder/en/.

[b0345] Yee N., Bailenson J. (2007). The Proteus effect: The effect of transformed self-representation on behavior. Human communication research.

[b0350] Zhang S., Han P., Wu C. (2019). A unified empirical likelihood approach for testing MCAR and subsequent estimation. Scandinavian Journal of Statistics.

