# Supplementary material for: Proteus effect avatar profiles: Associations with disordered gaming and activity levels
Source: Addict Behav Rep. 2024 Aug 2;20:100562. doi: 10.1016/j.abrep.2024.100562 (PMC11362772; doi:10.1016/j.abrep.2024.100562)
Supplement: Supplementary Data 1 [file mmc1.docx]

**Supplementary Table 1**

*Parameterization of Variance–Covariance Structures From the Most to the Least Restrictive Model.*

| Model | Variances | Covariances | Parameterization type |
| --- | --- | --- | --- |
| Class-invariant diagonal parameterization model (CIDP) | Equal | Fixed to 0 | This model assumes that relationships across model indicators should not be estimated (covariances fixed at zero) and that different profiles will be qualitatively similar (equal variances). |
| Class-varying diagonal parameterization model (CVDP) | Varying | Fixed to 0 | This model assumes that relationships between model indicators should not be estimated (covariances fixed at zero), and that different profiles will be qualitatively different (varying variances). |
| Class-invariant unrestricted parametrization model (CIUP) | Equal | Equal | Indicators are allowed to co-vary within profiles, and the variances and covariances are restricted to be equal across different profiles. |
| Class varying unrestricted parameterization (CVUP). | Varying | Varying | All the indicators are allowed to co-vary within profiles, and the variances and covariances (i.e., residual correlations) are allowed to be different across profiles. In other words, this model assumes that there are relationships between model indicators within and between latent profiles that should be estimated (i.e., varying covariances), and that different profiles will be qualitatively different (varying variances). |

*Note.* In this context, “diagonal” indicates that the sum of elements in the variance–covariance matrix equals zero, thus effectively preventing the model from estimating covariances between indicators.
